# Pituitary Tumorigenesis—Implications for Management

**DOI:** 10.3390/medicina59040812

**Published:** 2023-04-21

**Authors:** Rodanthi Vamvoukaki, Maria Chrysoulaki, Grigoria Betsi, Paraskevi Xekouki

**Affiliations:** Endocrinology and Diabetes Clinic, University Hospital of Heraklion, School of Medicine, University of Crete, 71500 Crete, Greece; rodoulavamv@gmail.com (R.V.); mariachrisoul@gmail.com (M.C.); raniabetsi1979@gmail.com (G.B.)

**Keywords:** pituitary neuroendocrine tumors (PitNETs), pituitary adenoma, pituitary tumorigenesis, pituitary pathogenesis, genetic alterations, molecular pathways

## Abstract

Pituitary neuroendocrine tumors (PitNETs), the third most common intracranial tumor, are mostly benign. However, some of them may display a more aggressive behavior, invading into the surrounding structures. While they may rarely metastasize, they may resist different treatment modalities. Several major advances in molecular biology in the past few years led to the discovery of the possible mechanisms involved in pituitary tumorigenesis with a possible therapeutic implication. The mutations in the different proteins involved in the Gsa/protein kinase A/c AMP signaling pathway are well-known and are responsible for many PitNETS, such as somatotropinomas and, in the context of syndromes, as the McCune–Albright syndrome, Carney complex, familiar isolated pituitary adenoma (FIPA), and X-linked acrogigantism (XLAG). The other pathways involved are the MAPK/ERK, PI3K/Akt, Wnt, and the most recently studied HIPPO pathways. Moreover, the mutations in several other tumor suppressor genes, such as *menin* and *CDKN1B*, are responsible for the MEN1 and MEN4 syndromes and succinate dehydrogenase (SDHx) in the context of the 3PAs syndrome. Furthermore, the pituitary stem cells and miRNAs hold an essential role in pituitary tumorigenesis and may represent new molecular targets for their diagnosis and treatment. This review aims to summarize the different cell signaling pathways and genes involved in pituitary tumorigenesis in an attempt to clarify their implications for diagnosis and management.

## 1. Introduction

Pituitary neuroendocrine tumors (PitNETs), or pituitary adenomas (PAs) as previously known, account for 15% of all intracranial tumors following gliomas and meningiomas with a mean incidence of approximately 5.1 cases per 100,000 per year [1,2]. The term PitNET has just been presented in World Health Organization’s (WHO) new classification to include their aggressive potential and highlight their neuroendocrine origin. Thus, we have chosen to endorse this terminology [3]. According to the data derived from the autopsy and radiological imaging series as well as the population studies, the observed frequency of the PitNETs in the general population is around 15–20%. However, most of these tumors are incidental findings with no apparent clinical impacts [4,5]. Metastatic PitNETs are rare (0.1–0.5% of cases) [6]. The rapid development and widespread use of neuroimaging technology, such as brain MRIs, enhanced methods of endocrine hormone determination, immunohistochemistry, and other technologies, have led to an increase in the detection rate of PitNETs, which piqued our interest into researching the pathogenesis of these tumors [2]. 

PitNETs can cause symptoms due to hormonal hypersecretion and/or the size and local mass effects suppressing the normal pituitary gland and surrounding tissues [7]. They are classified according to their size in microtumors (<1 cm), macrotumors (≥1 cm), or giant tumors (≥4 cm). Macrotumors (40% of PitNETs) are those that cause symptoms due to mass effects (pituitary insufficiency, bilateral hemianopia) or due to cavernous sinus infiltration [7]. Approx. two-thirds of PitNETs may secrete excess hormones. Lactotroph adenomas are the most common, accounting for 40% to 66% of the cases, followed by non-functioning PitNETs (14% to 43% of cases), somatotropinomas, corticotropinomas, and thyrotropinomas [2]. Non-functioning PitNETs (NF-PitNETs) are usually diagnosed later in contrast to the functioning ones, which are diagnosed earlier but are accompanied by a two to three times higher morbidity and mortality rate, as in the case of Cushing’s disease or acromegaly [8]. According to the 2022 WHO classification, the immunohistochemistry for the pituitary hormones and transcription factors that regulate differentiation is mandatory for the accurate classification (Pit1 lineage, Tpit lineage, SF1 lineage, no distinct cell lineage) and subclassification [3]. In accordance with this classification, features such as the rapid growth, imaging findings of the invasion of the surrounding tissues, and the high Ki-67 proliferation index, as well as the specific subtypes, such as the sparsely granulated somatotrophs, corticotrophs, lactotroph adenomas in men, immature Pit1 lineages, and silent corticotrophs, are associated with a more aggressive behavior [3].

Surgery is the first option for acromegaly and Cushing’s disease, especially when significant structures such as the optic chiasm are threatened. However, some PitNETs are successfully managed with agents targeting the somatostatin receptors 1–5 (SSTR1–5) and the dopamine agonist (DA) receptors. For acromegalic patients whose surgery has failed, or whose tumors are unresectable, the first-generation SSAs octreotide and lanreotide represent the first-line treatment, followed by the second-generation SSA pasireotide. However, approx. 50% of patients show a resistance to somatostatin analogs (SSA). The other treatment options represent the DA cabergoline, the GH receptor antagonist Pegvisomat, and in special cases, radiotherapy [1,9,10]. In lactotroph adenomas, DAs, cabergoline, and bromocriptine are quite effective for PRL normalization (85% of patients) and the reduction in the tumor size (80% of patients) and represent the treatment of choice for most patients. However, a minority of patients display a resistance to DAs exhibiting a more aggressive behavior and require different therapeutic modalities, such as high-dose cabergoline, surgery, radiation therapy, or temozolomide [11]. On the other hand, for NF-PitNETs, treatment using SSAs or DAs seems to have limited efficacy [12]. 

At present, PitNETs are considered to be of a monoclonal/oligoclonal origin due to somatic genetic mutations or chromosomal abnormalities (Table 1). Most of them are sporadic, and in 60% of cases, the somatic alterations of the oncogenes, tumor suppressor genes, and transcription factors regulating the cell growth and differentiation have been identified. Familial cases represent 5% of PitNEts, which are increasingly recognized as clinicians become more acquainted with familial syndromes, such as familial isolated pituitary adenomas (FIPA), multiple endocrine neoplasia types 1 and 4, X-linked acrogigantism, Carney complex, 3PAs, DICER1, and CABLES1 [13]. In the context of genetic syndromes, PitNETs appear at a younger age, have a larger size, a more aggressive behavior, and in some cases, are more resistant to treatment [14,15]. However, the specific mechanisms for many PitNETs are yet to be clarified. 

Different conserved signaling pathways, such as the MAPK, PI3K/Akt, and Wnt pathways, have been associated with pituitary tumorigenesis, while their regulation seems to be tissue-specific [16]. Recently, Hippo signaling has been linked to pituitary development and stem cell regulation, as well as poorly differentiated pituitary tumors [17]. Furthermore, pituitary stem cells have been identified in PitNETs, implying their crucial role in pituitary oncogenesis [18]. However, miRNAs seem to hold an essential role since they may provide new molecular targets for their diagnosis and treatment [19].

In the current review we summarized the already existing mechanisms contributing to the pathogenesis of the PitNETs in an attempt to understand their implications for management.

## 2. Cell Signaling Pathways in Pituitary Tumorigenesis

### 2.1. Gsa/Protein Kinase A/c AMP Signaling Pathway

**Figure 1:**
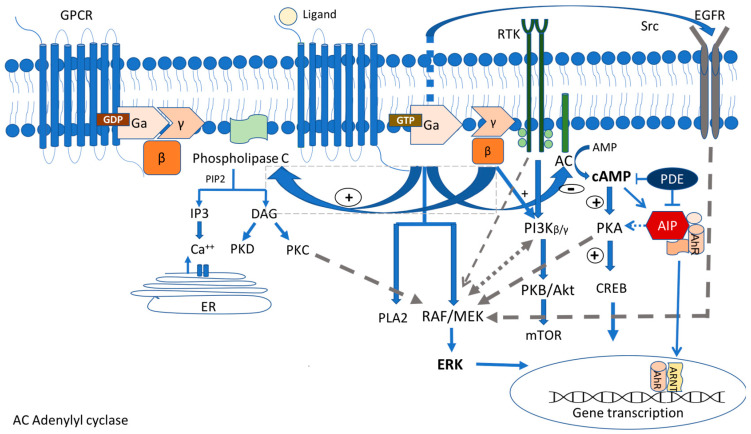
G-protein-coupled receptor (GPCR) signaling pathway. Interaction with the aryl hydrocarbon receptor-interacting protein (AIP), epithelial growth factor receptor (EGFR), and receptor of tyrosine kinase (RTK) signaling pathways, MAPK/ERK and PI3K/Akt pathways. Ga, Gβ, and Gγ are the subunits of the GPCRs. IP3: inositol triphosphate, DAG: diacylglycerol, PKD: protein kinase D, PKC: protein kinase C, PKA: protein kinase A, PI3Kβ/γ: phosphoinositide-3-kinase, ERK: extracellular signal-regulated kinase, platelet activating factor 2 (PLA2), m TOR: mammalian target of rapamycin, PDE: phosphodiesterase, AhR: aryl hydrocarbon receptor, ARNT: aryl hydrocarbon nuclear translocator.

The G-protein-coupled receptor (GPCR) signaling pathway represents one of the most crucial signaling cascades in development, normal physiology, and disease, and c-AMP, the major second messenger affected by this activation, is the first one described in [20]. GPCRs transmit extracellular signals mainly through heterotrimeric G proteins. Their molecule consists of three main subunits, Ga, Gβ, and Gγ (Figure 1). The Ga subunit is the one that defines the nature of each G-protein involved in the hormone action. It can be stimulating (Gs) (activating adenyl cyclase (AC) and increasing the cytosolic c AMP levels) and inhibitory (Gi/o/z) (inhibiting adenylyl cyclase, decreasing the intracellular cAMP levels, and regulating Ca and K as well). Moreover, it can act through the stimulation of phospholipase C Gaq(Gq/11) [21]. Nevertheless, these signaling pathways commonly overlap [22,23]. 

Whenever a ligand (i.e., a hormone) attaches to a GPCR, it results in a change in the conformation of the GPCR, the activation of the G protein, and the replacement of the GDP bound to the alpha subunit using GTP and leading to the dissociation of the other β- and γ- subunits. This process is accelerated by the regulators of G protein signaling (RGS) proteins. As a result Ga and the Gβ/γ heterodimer can act in different ways depending on the isoforms of the proteins [24]. Consequently, a downstream signaling cascade leads to the activation of the enzyme AC, which catalyzes the conversion of adenosine triphosphate (ATP) into cAMP and pyrophosphate [25]. This leads to the activation of protein kinase A (PKA), which is the main effector of the cAMP pathway and one of the best-studied kinases in human biology. PKA consists of two catalytic and two regulatory subunits. The binding of two molecules of cAMP to the regulator subunits leads to the dissociation of the catalytic subunits, which then translocate to the nucleus, permitting their serine–threonine kinase activity through binding the cAMP response element-binding protein (CREB) and triggering the transcription of several target genes [26]. In addition to the CREB, the PKA catalytic subunits can phosphorylate serine and threonine residues, such as many membrane, cytosolic, and nuclear target proteins, including phosphodiesterases (PDEs) and GPCRs, phospholipases, ion channels, histones, multiple protein kinases, phosphatases, and transcription factors [26]. cAMP is inactivated by PDEs by catalyzing the hydrolysis of cAMP and cGMP [27]. Moreover, GPCRs also interact directly with other ligands, such as JAK/STATs, Src-family tyrosine kinases, GRKs/b-arrestins, and PDZ domain-containing proteins, transducing the alternative G-protein-independent signals [28].

In the pituitary gland, the cAMP pathway is involved in cell proliferation, hormone synthesis and secretion, as well as tumor formation. The most common genetic alterations are described below [29]. 

#### 2.1.1. GNAS Mutations

The first mutation identified in pituitary tumors was in the GNAS gene [30]. The GNAS gene is located on human chromosome 20q13.13 and is one of the most frequently mutated genes in human tumors. The Gs-alpha subunit of the stimulatory G-protein is the best-studied product of the GNAS gene. Gs-alpha is expressed biallelically in many tissues and plays crucial roles in a plethora of physiologic processes. However, in a small number of tissues, such as proximal renal tubules, thyroid, gonads, and pituitary tissues, it is predominantly expressed from the maternal GNAS allele [31]. The other transcripts produced by GNAS are expressed exclusively from either the paternal or the maternal GNAS allele [32,33]. 

Somatic mutations in the GNAS gene, historically called gsp oncogene, are most frequently confirmed in growth hormone (GH)-secreting PitNETs, accounting for approx. 35–40% of sporadic tumors [34]. Additionally, the GNAS mutation, p.R201C was also detected in corticotropinoma [35] and in non-secreting PitNETs [36]. The most common mutations affect codon 201 or 227, leading to an aberrant GTPase activity, increased levels of cAMP production, increased PKA activity, and a constitutive phosphorylation of CREB. As a result, the somatotroph cells proliferate quickly and they show an uncontrolled GH synthesis and release [29]. These effects are counteracted by somatostatin, which binds the Gi/o protein complexes to SSTR1-5 [37]. The data regarding the behavior and response to the treatment of GNAS-mutated tumors is controversial. Many studies concluded that these tumors seemed to be smaller, grow slowly, be less likely to invade local tissues, and have a tendency to respond better to SSAs [38,39,40,41]. Moreover, recent a multiomic analysis of a GNAS locus in two independent somatotroph tumor cohorts revealed that 43% of gsp-negative tumors showed a GNAS imprinting relaxation, which corresponded to a lower GNAS, SSTR2 and AIP expression, and a lower sensitivity to SSAs and potentially aggressive behavior [42]. However, the Oxford cohort showed that the granulation pattern in a tumor subtype, not the gsp mutation, predicts the tumor response to the SSAs while a Brazilian cohort showed no difference between the tumors with or without a GNAS mutation [39,43]. To conclude, even though the prevailing opinion is that the GNAS mutation is an indicator of a better response to the treatment, there are studies with controversial results. 

The McCune–Albright syndrome (MAS) is a rare disease with an estimated prevalence between 1/100,000 and 1/1,000,000. It is characterized by the clinical trial of fibrous dysplasia of bone (FD), café au lait skin spots, and precocious puberty (PP). Other endocrine disorders may be involved, including hyperthyroidism, GH excess, Cushing syndrome, and renal phosphate wasting [44,45]. This is the result of postzygotic activating mutations of the GNAS1 gene product, Gs, with the vast majority consisting of point mutations at the Arg201 position. The syndrome is characterized by somatic mosaicism since the normal as well as the mutated cells can be identified throughout the body, indicating that the mutational event occurs early in embryonic life [46]. GH hypersecretion due to somatotroph hyperplasia or PitNETs is an uncommon manifestation of the MAS, affecting approx. 20% of patients, and is almost always accompanied by fibrous dysplasia of the skull. Since it is difficult to reach the pituitary gland in these patients, surgical treatment is not always an option. Most of them respond well to SSAs alone or with a combination of DAs. However, they show a much better response in the GH receptor antagonist pegvisomant [47,48]. Radiation treatment should be avoided as it may contribute to the malignant transformation of the dysplastic bone tissue [49]. Special attention should be paid when there is a co-existence of precautious puberty and GH excess in order to reduce the growth velocity and stabilize the bone age in these patients [50].

#### 2.1.2. Protein Kinase A Mutations

The Carney complex (CNC) is a rare genetic syndrome inherited in an autosomal dominant manner. In some cases, it occurs sporadically due to de novo mutations. It is characterized by the presence of multiple cardiac and extracardiac myxomas, spotty skin pigmentation, schwannomas and endocrine tumors, such as GH-secreting PitNETs, corticotroph tumors, and ACTH-independent Cushing syndrome known as primary pigmented nodular adrenocortical disease (PPNAD), and thyroid and gonadal tumors [51,52]. The mutations in the two loci were identified as 17q22-24 and 2p16, which contain the genes that are potentially responsible for the disease (initially known as CNC1 and CNC2). However, more than 70% of cases are due to heterozygous loss-of-function mutations in the PRKAR1A gene situated at the 24.2–24.3 locus of the long arm of chromosome 17, encoded for the regulatory subunit type I alpha of the PKA enzyme [51]. Most of these mutations are nonsense, frameshift, or splice site mutations leading to a defective mRNA [51,53]. The loss of the regulatory subunit 1 of PKA increases its responsiveness to cAMP, leading to uncontrolled somatotroph cell proliferation, as previously described [23]. The mutation in the *PRKACB* gene located at chromosome 1p31.1, which encodes the catalytic subunit beta of PKA, has also been identified in a patient with acromegaly, abnormal skin pigmentation, and myxomas Figure 1 [54].

Up to 75% of patients may have an asymptomatic elevation of GH, insulin growth factor I (IGF-1), and prolactin. Approx. 65% of them may exhibit somatomammotrophic hyperplasia (SH), while only 10–12% of them carry PitNETs [55,56], resulting in gigantism or acromegaly depending on the age of the presentation. Apart from acromegaly, there are some rare reports of lactotroph adenomas [57] as well as corticotroph tumors [58,59], although the ACTH-independent Cushing syndrome prevails in patients with the Carney complex. Acromegaly usually has a slowly-progressive course, and in most cases, it appears no earlier than 30 years old [52]. There is a phenotype—genotype correlation depending on the type of mutation of the PRKAR1A locus, where larger deletions may lead to a more severe phenotype [60,61]. Since these patients do not always carry an obvious PitNET, surgery may not always be a treatment option. The somatostatin analogs or GH receptor antagonists may be used either adjunctly to surgery or in patients with no detectable tumors [52]. 

#### 2.1.3. AIP

Familial isolated pituitary adenomas (FIPA), firstly recognized in 1999, are characterized by the presence of PitNETs in two or more members of the same family without other clinical features found in the context of a syndrome, such as in MEN1, MEN4, Carney complex, or succinate dehydrogenase (SDHx)-related tumors [62,63]. Approx. 20% of a FIPA harbor germline loss-of-function mutation in the aryl hydrocarbon receptor-interacting protein (*AIP*) gene map on the chromosome 11q13.3 locus [64]. However *AIP* mutations have been recognized in sporadic PitNETs, particularly those that occur during childhood/adolescence and early adulthood, probably explained by the incomplete penetrance of the disease (approx. 30%) [65,66]. AIP patients usually have macrotumors, with the first onset of symptoms occurring in childhood/adolescence in about 50% of patients. The most common type is a somatotroph tumor (50%), either as a pure GH secretor or as PRL-GH co-secretors, followed by lactotroph adenomas and NF-PitNETs, while corticotroph- and TSH-secreting tumors are quite rare [65]. Interestingly, different types of PitNETs may exist in a FIPA family and the clinical profile of the affected patients is variable, while pituitary apoplexy can also be a presenting feature [67].

The AIP is a co-chaperone protein that is expressed in many tissues and has a tumor suppressor function. It is able to bind to different partners using three antiparallel tetratricopeptide a-helix motifs (TPR domains), resulting in multiple protein–protein interactions [68]. The loss-of-function AIP mutations lead to a disruption of these interactions, probably contributing to pituitary tumorigenesis [64]. One of the most critical interactions is with PDEs, particularly PDE4A5, leading to decreased enzymatic activity and, therefore, negatively regulating the cAMP pathway in the pituitary gland [68,69]. However, the impact of the loss of this interaction in the context of an AIP mutation is still not completely understood and multiple post-receptor mechanisms and other signaling pathways are involved in pituitary tumorigenesis [70]. Moreover, the loss of function in the AIP leads to defective inhibitory GTP-binding protein (Gai) signaling. The Gai-2 protein levels seem to be reduced in the AIP-mutated somatotroph tumors [71]. Recently, it was shown that the AIP interacts with the main regulatory (R1a) and catalytic (Ca) PKA subunits, providing novel insight into the involvement of the AIP in the cAMP pathway tumorigenesis [72]. 

In addition to the involvement in the c AMP pathway, the AIP exerts its effects by binding and stabilizing the aryl hydrocarbon receptor (AhR), which is best known for mediating the effects of environmental toxins, such as dioxin, the so-called “dioxin receptor”. The AhR is a member of the basic helix-loop-helix/Per-Arnt-Sim (bHLH/PAS) family of transcription factors that regulates the response to halogenated hydrocarbons. It is involved in different cell responses and the regulation of the cell cycle and differentiation. In the cytoplasm, it is stabilized by forming a multimeric AIP/AhR/Hsp90/p23 complex [68], avoiding the AhR degradation. Upon ligand binding, the AhR disengages and translocates to the nucleus, where it binds to the aryl hydrocarbon nuclear translocator (ARNT). Together with several co-activators and co-repressors, the AhR forms a transcription complex on the DNA sequences, called xenobiotic response elements (XRE), leading to the transcription of the relevant genes (Figure 1) [73]. The exact mechanism by which the AhR is involved in tumorigenesis is not fully understood. However, it was shown that the AIP mutations resulted in decreased AIP expression and altered the AhR transcriptional response in human fibroblasts [74]. Furthermore, the AhR seems to have a putative tumor suppressive role in PitNETs [75]. Another AIP interactor is the zinc finger protein 1(ZAC1), which acts as transcription factor and coregulator in the pituitary cells and plays an important role in pituitary tumorigenesis [76].

AIP-mutated pituitary tumors have a broad clinical spectrum. GH-secreting PitNETs usually have an aggressive profile, higher levels of GH and IGF1, and show a resistance to the treatment using first-generation SSAs-octreotide and lanreotide [65]. Thus, a low AIP tumor expression is an indicator of tumor aggressiveness and treatment resistance [77]. Chahal et al. suggested that octreotide may increase the expression of the tumor suppressor gene ZAC1, and the loss of expression of ZAC1 occurring in AIP-mutated adenomas results in an SSA resistance [78]. Dutta et al. reported a four-year-old child with an AIP pituitary macrotumor, which required multimodal treatment with surgery, long-acting octreotide, radiotherapy, temozolomide, bevacizumab, and pegvisomant to be controlled [79]. However, not all AIP-mutated patients are resistant to octreotide. Some patients may present indolent PitNETs detected by screening tests in mutation carriers, who might have a good response to standard treatments [80]. A number of patients with AIP-mutated gigantism has been described in the literature with a good response to the combined treatment using SSAs and pegvisomant [81]. In an observational retrospective study performed in 77 AIP-negative acromegalic patients, who were screened for both the AHR rs2066853 variant and the glutathione-S-transferase-P1 (GSTP1) gene promoter methylation, those with the methylated GSTP1 gene promoter were found to be more resistant to SSAs. The patients with the non-methylated GSTP1 and the AHR wild-type were the most sensitive to SSA treatment, while those with both the GSTP1-methyl and the AHR rs2066853 variant were resistant to SSAs [82]. Nevertheless, there are some reported AIP patients who responded better to pasireotide [83,84]. Moreover, it was recently demonstrated that AIP might act in the initial steps of the RET-apoptotic pathway in the Pit1-expressing cells, and a lack of its expression promotes the Ret survival pathway, leading to tumorigenesis. This new interplay between the AIP and the RET signaling pathway adds impetus to the research in AIP aggressive pituitary tumors and may constitute an alternative target for new therapeutic approaches [85]. Another potential therapeutic target could be the tumor-derived cytokine CCL5, which seems to be upregulated in AIP-mutation-positive human adenomas. The inhibition of the CCL5/CCR5 pathway by maravirorik was efficiently proven using mice experiments. Hence, the crosstalk between the tumor and its microenvironment might play a key role in the invasive nature of AIP-mutation-positive tumors [86]. 

The genetic and clinical screening for AIP mutations is significant since earlier diagnoses show better outcomes than the clinically presenting cases [87]. 

#### 2.1.4. GPR101

The second known cause of FIPA is due to the germline or somatic microduplication in chromosome Xq26.3, which includes the orphan G-protein-coupled receptor (GPCR) gene, GPR101, a copy number variation (CNV) that is responsible for the so-called X-linked acrogigantism (X-LAG) syndrome first described in 2014 by Trivellin et al. [88]. However, there are also sporadic cases that were detected. The c.924G > C (p.E308D) *GPR101* missense variant was identified in 4.4% of a series of patients with sporadic acromegaly [89]. In the cases that were reported so far, the duplications were germline in females whereas they were somatic in sporadic males with variable levels of mosaicism [90,91]. However, both sexes had a similar phenotype. These patients were characterized by early childhood (<5 years old in most cases) onset gigantism due to GH-secreting tumors, mixed GH- and PRL-secreting (85% of cases) PitNETs, or hyperplasia [92]. There is evidence that pituitary hyperplasia precedes tumor formation in XLAG patients [88].The disease has a female predominance and female patients seem to be younger—as young as two months old—than males. Moreover, apart from the acromegalic features and growth acceleration, they tend to have acanthosis nigricans, insulin resistance, and an increased appetite, probably explained by the GPR101 expression at the hypothalamus [93].

GPR101 encodes a class A, rhodopsin-like orphan GPCR coupled to Gs subunit. Until now, no ligand has been identified as being responsible for the pituitary tumor formation [94]. This receptor is normally expressed at the hypothalamus, the nucleus accumbens, and the pituitary gland during fetal life and adolescence. However, relative, scarce, or absent expression is detected during childhood and adult life [89,95]. The duplication of *GPR101* probably affects the GH secretion both at the pituitary and the hypothalamic level. In pituitary tumors harboring a GPR101 duplication, even in the absence of a ligand, the overexpressed GPR101 receptor interacts with the cAMP pathway leading to its constitutive activation and triggering a sequela of proliferative events [88,96]. However, in one study, it was shown that GPR101 did not constitutively activate the cAMP pathway, while in the same study, GPR101 was also found to inhibit the forskolin-stimulated CRE reporter activity, supporting the fact that it might bind to both stimulatory (Gs) and inhibitory (Gi) proteins [97]. Moreover, recent studies in mice showed that GPR101 can potentially activate Gq/11 and G12/1, leading to elevated levels of GH but not somatotroph hyperplasia and proliferation [98]. Moreover, GnRH-(1-5), a pentapeptide derived from the decapeptide gonadotrophin-releasing hormone (GnRH), was recently reported as a potential binder for GPR101. This connection induces the epidermal growth factor (EGF) release, followed by the EGF receptor (EGFR) phosphorylation with a consequent activation of the downstream signaling pathways, leading to increased cell proliferation. The latter was detected in endometrial cancer cells. However, how this ligand affects the pituitary cells was not shown [99]. In addition, the XLAG patients were found to have elevated circulating GHRH levels, leading to a further stimulation of GH and the prolactin secretion. Furthermore, the dysregulation of the GHRH secretion was in accordance with the hypothalamic GPR101 expression, which may have further contributed to the abnormal pituitary cell proliferation [92,96].

Although there is high expression of SST2 receptors in XLAG tumors, they are resistant to medical treatments using SSAs and DAs [93,96]. Therefore, in most cases, surgery seems to be the best approach with near total hypophysectomy, followed by additional pegvisomant treatment if there is residual disease [93,100]. Interestingly, the therapeutic blockade of the GHRH secretion may represent a potential new therapeutic approach in the XLAG syndrome, which adds impetus to the research in this field [92].

### 2.2. MAPK/ERK and PI3K/Akt Pathways

**Figure 2:**
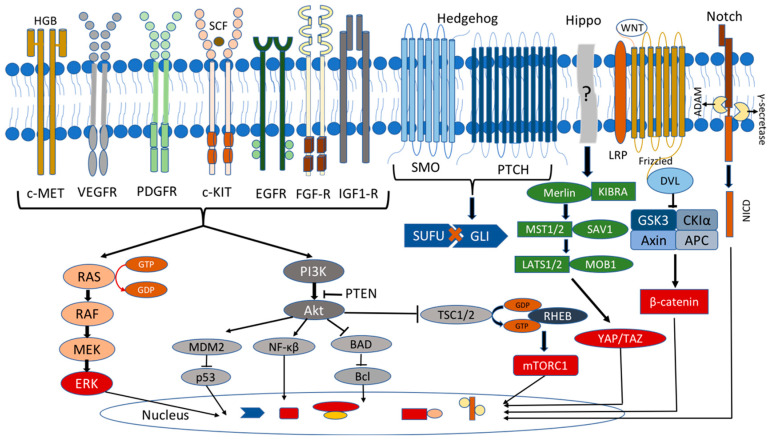
Molecular pathways involved in pituitary tumorigenesis. Growth factor signaling (receptor tyrosine kinases e.g., MET [103], KIT [104], PDGF [105], IGF-1, EGF, VEGF, and FGF receptor families [106]), Hippo signaling [17,107], Wnt signaling [108], Notch signaling [109], and Hedgehog signaling [110]. c-Met: tyrosine-protein kinase Met; HGB: hemoglobin; c-KIT: tyrosine-protein kinase KIT; SCF: stem cell factor; PDGFR: platelet-derived growth factor receptor; ERK: extracellular signal-regulated kinase; MDM1: the mouse double minute 2; BAD: BCL2-associated agonist of cell death; TSC: tuberous sclerosis complex1/2; RHEB: Ras homolog enriched in brain; mTORC: mechanistic target of rapamycin; SMO: smoothened; PTCH: protein patched homolog 1; SUFU: suppressor of fused homolog; Gli: glioma-associated oncogene homologue; MST1/2: mammalian sterile 20-like 1/2; SAV1: salvador; LATS1/2: large tumor suppressor homolog 1/2; MOB1: MOB kinase activator 1; YAP: yes-associated protein, TAZ: transcriptional co-activator with PDZ-binding motif (also called WWTR1); LRP: lipoprotein receptor-related protein; DVL: disheveled; GSK-3β: glycogen synthase kinase-3β; APC: adenomatosis polyposis coli; CK1α: casein kinase 1 alpha; NICD: intracellular domain of the Notch protein; ?: diverse, and some of them still unspecified upstream signals.

The mitogen-activated protein kinase (MAPK) signaling pathway regulates a variety of physiological processes, such as cell growth, differentiation, and apoptosis, and has been linked to many types of tumors, including lung, prostate, and colorectal cancers [101,102]. In the MAPK pathway, GTPase Ras is activated by several extracellular growth factors and mitogens after binding to the receptor tyrosine kinases (RTKs) (e.g., IGF-1, EGF, VEGF and FGF receptor families) and the G-protein-coupled receptors (GPCRs). The activated Ras stimulates the protein kinase Raf to phosphorylate and activate MEK and ERK1/2 kinases, which phosphorylate numerous cytoplasmic and nuclear targets, including kinases, phosphatases, and transcription factors (Figure 2). The sustained Ras/ERK signaling has been linked to the upregulation of the genes required for the cell cycle, such as cyclin D1, and the repression of the expression of the genes that inhibit the proliferation, leading to uncontrolled cell proliferation and tumorigenesis [101,102]. 

It is well established that the MAPK signaling pathway is involved in PitNETs. Two cases of lactotroph adenomas have been found to harbor H-Ras mutations [111,112]. The overexpression of the B-Raf mRNA and protein is a predominant finding in NF-PitNETs [113]. Moreover, the downstream components of B-Raf were also over-activated in these tumors [114]. The experimental studies in mice showed that the outcome of the MAPK pathway is pituitary cell-type specific. In the lactotroph cells, the precise role of the ERK signaling on the cell proliferation depended on the exposure time of the activation. A short-time activation of the ERK (24–96 h) enhanced the in vitro proliferation of the rat pituitary lactotroph or somatolactotroph cell lines [115,116]. Contrary to this finding, when the ERK signaling was activated for a long time (over 6 days) the somatolactotrope cells were differentiated into a lactotroph cell phenotype characterized by a decreased proliferation and tumorigenicity [117]. Similarly, in thyrotropes, the ERK pathway had antiproliferative effects. Treating thyrotropinomas with thyroid hormones (THs) activated the ERK pathway and prevented cell proliferation and tumor growth whereas the TH withdrawal reversed this action [118]. In the somatotroph cells, the ERK signaling was well documented to have a pro-proliferative efficacy through the receptor of GHRH [119]. Lania et al. showed that protein kinase C (PKC) activated ERK1/2 and increased the cell proliferation through the GHRH receptor whereas PKA activated ERK1/2 through the cAMP pathway in a receptor-independent manner [120]. The ERK signaling was also responsible for the GH production by somatotrophs [121]. SSAs, the gold standard therapy for somatotroph tumors, have been demonstrated to inhibit the ERK signaling in patients independent of the expression profile of the SSTRs [122]. In the gonadotroph cells, GnRH activated the members of the MAPK family (ERK, JNK, and p38 MAPK), contributing to the expression of the luteinizing hormone (LH), and ERK was activated via a PKC-dependent pathway [123]. NF-PitNETs, which for the majority were gonadotroph in origin, were characterized by the B-Raf overexpression and ERK activation compared to the normal pituitaries [113,124]. Regarding the corticotroph cells, the ERK signaling had pro-proliferative effects. The treatment using a MEK inhibitor binimetinib, both in vitro and in vivo, inhibited the corticotroph tumor cell proliferation, POMC transcription, and ACTH secretion, rendering it a possible candidate for Cushing’s disease treatment [125].

The BRAF protein, a member of the RAF kinase family, is characteristically mutated in papillary craniopharyngiomas (PCPs) with the gain-of-function mutation BRAFV600E found in most PCPs. Although it was thought that such mutation was exclusively found in PCPs, it was recently reported to coexist with CTNNB1 in adamantinomatous craniopharyngiomas (ACPs) [126,127]. Interestingly, the MAPK pathway plays a crucial role in controlling the stemness in embryonic as well as adult stem cells. The constative activation of the pathway leads to a high-proliferative rate of the SOX2 + cells, and it has been implicated in maintaining an undifferentiated tumorigenic state, which may underlie the pathogenesis of PCP [128].

There is rising evidence about the use of BRAF inhibitors (vemurafenib, dabrafenib) as monotherapy [129,130,131,132] or in combination with the MEK inhibitors (cobimetinib and trametinib), as reported in patients with recurrent/progressive BRAFV600E-mutated PCPs with a majority of favorable results [133,134,135]. Moreover, the use of BRAF/MEK inhibitors has been proposed as a neoadjuvant treatment for surgery, radiosurgery, or radiotherapy [134,135,136]. The clinical trials that are currently evaluating the drug targets in craniopharyngiomas are limited, and only one is studying the treatment of BRAFV600E mutant PCPs (ClinicalTrials.gov identifier (NCT number): NCT03224767). This phase II clinical trial examines the combination therapy with a BRAF inhibitor (vemurafenib) and a MEK inhibitor (cobimetinib) in adults 18 years or older with previously untreated BRAFV600E PCP [137]. To date, the results are encouraging, as 15 out of 16 patients responded to the combined therapy with vemurafenib/cobimetinib, and only one patient did not respond at all because the treatment was discontinued earlier due to toxicity [137]. Therefore, this study provides evidence that BRAF/MEK inhibitors might be a good option for the treatment of previously untreated PCP. However, there is no doubt that their use should be evaluated in further studies with more patients enrolled. At the moment, a second arm of this study is evaluating patients with progressive PCP after prior radiotherapy [137].

MAPK signaling is a complex multi-network as it is now established that it interacts with other pathways, such as PI3K/AKT/mTOR and the cAMP pathway, to affect tumorigenesis [138,139,140]. The PI3K/AKT/mTOR signaling pathway is traditionally involved in cellular functions, such as cell growth, proliferation, differentiation, motility, survival, and cancer. This pathway is activated by receptor tyrosine kinases (RTKs), leading to the auto-phosphorylation of the receptor and PI3K allosterically activation, resulting in the conversion of PIP2 to PIP3. PIP3 binds to the pleckstrin homology domain of AKT, facilitating the phosphorylation of AKT using phosphoinositide-dependent kinase-1 (PDK1) and mTORC2 (Figure 1 and Figure 2). Phosphorylated AKT is active and can phosphorylate mTORC1 or other effectors to regulate the normal cell proliferation [101,102]. Moreover, the PI3K/AKT/mTOR pathway is regulated by the tumor suppressor PTEN, a phosphatase that dephosphorylates PIP3 and inhibits this pathway [141]. 

Several studies have examined the relationship between the PI3K/AKT/mTOR pathway and pituitary tumorigenesis. In a novel study, Chen et al. generated Pit1 lineage-specific mTOR-activated mice that developed lactotroph adenomas by 14 months. In addition, they demonstrated that the mTOR activation caused lactotroph adenoma in the mice by activating the pituitary tumor transforming gene 1 (PTTG1) [142]. Adding to these data, cabergoline, a first-line treatment for lactotroph adenomas, was recently shown to suppress the prolactin hypersecretion and reduce the tumor size via the mTOR inhibition pathway in a rat pituitary tumor [143]. Moreover, in a mouse model of TSH-secreting PitNETs, the AKT, mTOR, and the downstream effector p70s6K were activated, which led to an increased cell proliferation and reduced apoptosis [144]. Similarly, in corticotrope cells, the simultaneous inhibition of the PI3K/AKT/mTOR and Ras/ERK pathways contributed to a decreased cell proliferation and the inhibition of the POMC transcription, suggesting the involvement of PI3K/AKT/mTOR in pituitary tumorigenesis as well the therapeutic use of inhibitors targeting this pathway [145]. 

In human NF-PitNETs, the overexpression of AKT was noted in the absence of a PTEN mutation compared to the normal pituitary response [146]. Moreover, it was suggested that mTOR is activated in human GH-secreting PitNETs in an AKT-independent manner, although the mechanism has not yet been described in detail [147]. Consistent with the role of PI3K activation in PitNETs, the mutations of the PIK3CA gene that encodes the p110 catalytic subunit of PI3K were assessed in 353 pituitary tumors. The somatic mutations of the PIK3CA gene were detected in about 9% of invasive pituitary tumors but were note detected in any of the non-invasive tumors, and the mutation was associated with an increased disease recurrence [148]. Another study examined 33 PitNETs for PIK3CA mutations and showed that PIK3CA mutations were present in 12.1% of tumors, including one non-invasive ACTH tumor [149].

SSAs used for the treatment of PitNETs decrease the cell proliferation and inhibit the release of the growth factors and angiogenesis [150]. They exert their action through GPCRs, which are variably expressed in both normal pituitaries and PitNETs. The analog octreotide can activate the SST receptor subtype-2 (SSTR2) and SSTR5 with a lower affinity, while pasireotide (SOM230) can activate SSTR1, 2, 3, and 5 [151,152]. Notably, a couple of studies claimed that the inactivation of the ERK signaling was responsible for the antiproliferative effect of the SSAs; octreotide inhibited both the ERK and PI3K/Akt pathways while pasireotide mediated the ERK pathway [122,153]. Dopamine, which suppresses the PRL gene transcription and lactotroph proliferation, was reported to exert its action via the inhibition of the cAMP/PKA and MAPK pathways. Dopamine mediates the lactotroph homeostasis through the GPCR dopamine D2 receptor (DRD2) [154] and lactotrophs seem to express two isoforms of DRD2, D2L and D2S. However, the two D2R isoforms have been linked to independent transduction pathways, which have different roles in the pituitary gland physiology. The D2S isoform seems to decrease PRL and inhibit the lactotroph cell proliferation by stimulating the ERK signaling, while the D2L isoform has been shown to enhance the PRL secretion [155,156]. Since the current pharmacological treatments for PitNETs (SSAs, DA, or their combination) are unsuccessful in several patients, novel chimeric somatostatin/dopamine (dopastatin) compounds have been developed. It has been demonstrated that the somatostatin and dopamine receptors could heterodimerize to form a novel receptor with an increased functional activity, indicating a molecular cross-talking between the related G-protein-coupled receptor subfamilies [157]. Thus, based on this study, dopastatins were developed (BIM-23A387 and BIM-23A760), showing a reduced GH and PRL secretion in the primary cultures of somatotropinomas, especially in the tumors with a partial response to octreotide and lanreotide [158,159]. However, the later BIM-23A760 has been withdrawn from clinical development since the in vivo data demonstrated that its former metabolite had a higher dopaminergic activity than the parent compound, which interfered with it [160]. Thus, another chimeric somatostatin/dopamine compound, BIM-065, was recently designed with the same affinity to bind SSTR2, a higher affinity to bind SSTR5, and a slightly smaller affinity to bind DRD2 compared to BIM-23A760. Vazquez-Borrego et al. showed an increased apoptosis and the inhibition of the GH secretion in the primary cultures of somatotropinomas with an enhanced effect on the p-ERK1/2 and p-Akt levels [161]. Importantly, the inhibitory effect of BIM-065 on the GH secretion was higher compared to octreotide or pasireotide [161]. In line with these in vitro results, the treatment of rat somatotropinomas with BIM-065 resulted in a significant decrease in the tumor size after 4 weeks [162].

Regarding the PI3K/Akt/mTOR pathway inhibitors, the tumors that carry upstream mutations from mTOR, such as the PTEN deletion or AKT overexpression, are an ideal target. To date, temsirolimus and everolimus are the only FDA-approved mTOR inhibitors and are used for kidney or breast cancer [163]. Everolimus (RAD001), an oral analog to rapamycin, is the only active mTOR inhibitor administered in patients with PitNETs. A recent review summarized six cases treated using everolimus with favorable results in only one patient who was not previously treated with temozolomide [164]. Everolimus has been demonstrated to have anti-cancer effects in a number of in vitro cell lines as well as in mouse models [165,166,167,168]. It binds to the FKBP12 protein to inhibit mTOR, which results in a reduced protein synthesis, the inhibition of the cell proliferation, and the G0/G1 cell cycle arrest [166]. In vitro studies from human NF-PitNETs demonstrated that the combination of everolimus with an SSA (octreotide or pasireotide) results in a greater antiproliferative response than each drug individually [167,168]. Similarly, in the mouse pituitary corticotrope tumor cell line AtT-20, the co-administration with octreotide had the same results [168]. Additionally, the in vitro studies in the cell lines and in the human PitNETs assessed the use of PI3K inhibitors combined with mTOR inhibitors, demonstrating an enhanced mTOR inhibitor effect in the cell proliferation [164,169,170]. Notably, Day and his team underlined that the use of a novel dual-PI3K/mTOR inhibitor (XL765) in the pituitary cell lines and in the GH3 xenograft tumor model increases the anti-tumoral effect of temozolomide [171]. These data support the notion that the inhibition of PI3K and mTOR could be a promising therapeutic option for the treatment of aggressive PitNETs. Currently, there are many clinical trials that evaluate the use of the PI3K and mTOR inhibitors in different cancer types, and some are FDA-approved. However, no dual-PI3K/mTOR inhibitor is clinically available due to adverse effects [172].

As previously discussed, RTKs activate the MAPK/ERK and PI3K/Akt pathways leading to pituitary tumorigenesis. Therefore, the TK inhibitors could be an emerging therapeutic option. To date, the main TK target for the PitNETs is the epidermal growth factor family of the receptor tyrosine kinases (ErbBs), which consists of ErbB1 (called EGFR or HER1), ErbB2 (or HER2/Neu), ErbB3 (or HER3), and ErbB4 (or HER4). Ben-Shlomo and Cooper recently showed the POMC mRNA suppression after the treatment with gefitinib, an EGFR inhibitor, in both human and mouse corticotroph primary cultures [173]. In line with these findings, the use of lapatinib, a dual-EGFR and HER2 inhibitor, decreased the ACTH levels and inhibited the cell proliferation of the AtT-20 mouse corticotroph tumor cells [174]. Additionally, lapatinib seemed to have a more influential role in prolactin-secreting PitNETs than gefitinib both in vitro and in vivo, as it attenuated the PRL secretion and cell proliferation to a greater degree [175]. These findings indicated that HER2/ErbB2 induced the PRL and tumorigenic effect in the rat prolactin-secreting PitNETs. Similarly, in human studies, in which the expression of the ErbB receptors was correlated with the clinical tumor behavior, revealed that ErbB2 was mainly associated with aggressive and/or resistant prolactin-secreting PitNETs [176]. Moreover, a phase 2a clinical trial suggested that lapatinib may be a suitable treatment option for aggressive prolactin-secreting PitNETs. However, due to the small number of patients, a larger cohort size is required [177].

#### Ubiquitin-Specific Protease 8 (USP8) and Other Deubiquitinases

Ubiquitin-specific protease 8 (USP8) is a deubiquitinating enzyme (DUB) that plays an important role in enhancing cell proliferation and promoting the cells to enter the S-phase during the cell cycle [178].

The EGFR among the different RTKs is the most important protein, which is very frequently deubiquitinated and stabilized by USP8, and thus, promotes the initiation, progression, and metastasis of cancer by activating the numerous downstream signaling pathways, including HES 1, p21, c-Myc, E2F1, ACTH, STAT3, ERK, and pAKt, as well as inhibiting the tumor suppressor p53 and other apoptotic proteins [179].

Until recently, the tumorigenesis of Cushing’s disease was unclear. Reincke and colleagues revealed frequent somatic hotspot gain-of-function mutations (GOF) in the gene encoding for USP8, especially in female adult patients diagnosed at a younger age with a smaller tumor size [180,181,182]. The prevalence of somatic hotspot USP8 mutations ranged between 20% and 60% of the sporadic corticotroph tumors and were associated with a higher incidence of surgical recurrence significantly earlier than the wild-type tumors [182,183]. Recent cohort studies demonstrated that patients with USP8 mutant tumors had higher postoperative 24h-hour urinary-free cortisol and ACTH levels, indicating tumor recurrence [182,183]. Contrary to these data, a greater probability of surgical remission was reported by Hayashi et al., (mutant group (95.2%) vs. WT group (53.8%)) [184] while Ma et al. showed that the recurrence rate was unrelated to the USP8 mutational status [185]. Importantly, the heterogeneity of the USP8 clinical phenotype after transsphenoidal surgery could be attributed to the fact that the tumor size was generally associated with the clinical remission. However, in each situation, it was almost impossible to completely remove the tumor. Additionally, somatic USP8 mutations were also present in the Nelson syndrome. Almost half of Nelson’s tumors in a cohort of 33 cases contained a mutation in Ser718 or Pro720 [186]. USP8 mutations have only been found in corticotroph tumors, even though USP8 is expressed in all anterior pituitary cell types [185].

USP8 mutations are correlated using the overexpression of EGFR in ACTH-secreting PitNETs, and EGFR signaling has been recently suggested as a critical pathway for corticotroph tumorigenesis [180,181]. All the identified USP8 mutations are clustered in the 14-3-3 protein-binding motif in exon 14, a domain highly conserved across many species. Thus, the lack of the 14-3-3 binding motif enhances the proteolytic cleavage and DUB activity of USP8. To date, the S718del, P720R, S718P, and P720Q mutations are the most common alterations found in the USP8-mutated corticotroph tumors [180,185]. Interestingly, a de novo germline defect of USP8 (S719P) was reported in a young female patient with Cushing’s disease [187]. It was proposed that the USP8 protein product controls the lysosomal trafficking and the abundance of the cell surface receptors, such as EGFR, and that mutated USP8 enables their recycling and signaling [180,181]. Therefore, the high EGFR levels stimulate the *POMC* gene transcription, enhancing the ACTH synthesis. The inhibition of USP8 could be a promising treatment for Cushing’s disease. Indeed, chemical compounds such as compound 9-oxo-9H-indeno [1,2-b]pyrazine-2,3-dicarbonitrile (DUBs-IN-2) and RA-9 have been described as USP8 inhibitors in the experimental models of corticotropinomas. It has been demonstrated that the aforementioned USP8 inhibitors decreased the POMC mRNA levels and the ACTH levels in the murine AtT-20 corticotroph tumor cells and also inhibited the cell proliferation and induced apoptosis [188,189]. Thus, based on these favorable results on the AtT-20 cells, the blockade of USP8 might be useful for the treatment of human corticotropinomas, while further research should be conducted.

Pasireotide, an SSA with a high affinity for SSTR5, is the only pituitary tumor target drug approved for the treatment of Cushing’s disease [184]. The USP8 mutational status has been correlated with a higher SSTR5 expression and suggests a potential favorable response to pasireotide [184]. A novel study validated this hypothesis in vitro using human and murine corticotroph tumors overexpressing the human USP8 mutants. They showed that pasireotide exerts a higher antisecretory response of ACTH in the USP8-mutant corticotroph tumors [190]. Similarly, Treppiedi and her colleagues demonstrated that USP8 mutations were associated with an increase in the SSTR5 expression. However, they suggested that the pasireotide efficacy depends on the USP8 residue involved, as pasireotide could reduce the ACTH production only on the P720R USP8-mutated cells [191]. Therefore, the USP8 mutational status could be a potential marker of the pasireotide response.

Different whole exome sequencing studies revealed additional mutations in the deubiquitinase USP48, the BRAF oncogene, the glucocorticoid receptor NR3C1, and TP53 in USP8 wild-type corticotroph tumors, though at much lower rates [192,193]. Mutations in the deubiquitinase USP48 (p.M415I or p.M415V) were identified in 23% of corticotroph tumors with wild-type USP8, while the pathogenetic mechanism involved the NF-κB pathway, which is implicated in the CRH-induced transcriptional activation of the POMC gene [192]. Moreover, the same study revealed the somatic mutation V600E in BRAF in 16.4% of the cases, which enhanced the promoter activity and the transcription of POMC through the MAPK activation [192]. They also reported that primary corticotroph tumor cells harboring BRAF V600E were sensitive to the BRAF inhibitor vemurafenib, indicating its potential efficacy in the treatment of corticotroph tumors with the BRAF V600E mutation [192]. Similar to USP8, neither the BRAF nor the USP48 mutations were identified in the other PitNETs except for corticotroph tumors, suggesting their specificity and their essential role as a drug target.

### 2.3. Hippo Pathway

Initially described in Drosophila and highly conserved in mammals, the Hippo signaling pathway has been linked to diverse physiological and pathological processes. It is expressed early in fetal development and controls the organ size, homeostasis, and regeneration. However, it is also related to pathological processes, including cancer [194]. Recently, Lodge and colleagues showed that the Hippo pathway is active and necessary during embryonic development, including in human and mouse pituitary development [107,195]. The core mammalian Hippo pathway consists of a kinase cascade in which MST1/2 kinases phosphorylate and activate LATS1/2 kinases, which in turn phosphorylate the co-activators YAPs and TAZs that are subsequently inactivated through cytoplasmic retention via 14-3-3 binding or ubiquitinated and degraded (Figure 2). The nuclear active YAP/TAZs act as co-activators for the TEAD transcription factors, which are associated with growth, survival, and stemness [196]. Several lines of evidence indicated high levels and a nuclear localization of the YAPs/TAZs in many human tumors, such as liver, breast, lung, colon, pancreas, ovary, prostate, and others. They seemed crucial for cancer initiation, progression, metastasis, and drug resistance [197,198].

There is increasing evidence that the Hippo pathway plays a functional role in the pituitary gland, though it is strongly associated with the stem cell state. Pituitary stem cells are able to give rise to all endocrine cell types of the anterior pituitary gland and their dysregulation can lead to tumorigenesis [18]. It has been shown that the stem cell transcription factor SOX2 + interferes with the tumor suppressive Hippo pathway, leading to high YAP function and the repression of the differentiated state in the cancer stem cells in osteosarcomas [199]. The role of the Hippo pathway in pituitary development and stem cell regulation was shown for the first time by Lodge and her colleagues [107,195]. They found that the YAP and TAZ were active and primarily localized in the nucleus in SOX2 + pituitary stem cells throughout the development and at the postnatal stages in mice [195]. Subsequently, in a preliminary study, Xekouki et al., showed evidence of an immunohistochemical expression of the YAP/TAZ in fetal and adult human pituitary cells as well as an increased expression in the poorly differentiated pituitary tumors (null cell adenomas, ACPs and PCPs), and all tumors with a large undifferentiated compartment [17]. Consistent with the previous mouse data where the absence of LATS1 resulted in anterior pituitary hyperplasia and decreased the serum levels of GH, LH, and PRL [200], the knockdown of LATS1 in the rat GH3 mammosomatotropinoma cells repressed the GH and PRL promoter activity, further supporting the role of the Hippo dysregulation in pituitary tumorigenesis [17]. Furthermore, the postnatal deletion of LATS1 and the subsequent upregulation of the YAP/TAZ promoted the uncontrolled growth of the SOX2 pituitary stem cells and tumor formation, resembling pituitary cancer [107]. These in vitro and in vivo data support the notion that high levels of the YAP/TAZ may be associated with the maintenance of an active pituitary stem cell state during development as well as the inhibition of the differentiation. Thus, the characterization of the YAP/TAZ pattern could have a prognostic value and may be attractive targets for new treatments for pituitary tumors.

In a novel study, the generation of gonadotrope-specific YAP/TAZ conditional knockout mice *(Yap*^flox/flox^; *Taz*^flox/flox^; *Gnrhr*^GRIC/+^) was linked to the increased circulating levels of the luteinizing hormone (LH) in both male and female mice without affecting the GnRH signaling. The pharmacologic inhibition of the YAP/TAZ function using verteporfin (YAP1/TAZ-TEAD interaction inhibitor) in the immortalized gonadotrope-like cell line LβT2 revealed the same results. The circulating LH levels were increased but without affecting the Lhb expression [201]. These data suggest that the YAP/TAZ may have a negative regulatory role in the LH secretion machinery in gonadotrope cells without affecting the gonadotropin synthesis.

The YAP/TAZ signaling pathway has also been involved in a resistance to cancer therapy in several tumors. Emerging evidence indicates the activation of the YAP/TAZ in response to a pharmacological EGFR and RAS/MAPK inhibition, which acts as a bypass mechanism for the activation of alternative Hippo transcriptional target survival genes, such as AXL, Bcl-xL, CTGF, CYR61 [198,202,203]. Thus, the YAP/TAZ inhibitors could have a promising contribution for overcoming therapy resistance induced by the YAP/TAZ activation [204,205]. Several agents targeting the Hippo signaling components (such as verteporfin, metformin, statin, super TDU, CA3, etc.) have been described for their favorable effect on the different types of cancer in the experimental models [206]. Verteporfin (VP), an FDA-approved drug for treating wet aged macular degeneration, is the first YAP/TAZ–TEAD interaction inhibitor identified to suppress the YAP oncogenic activity and liver tumorigenesis [207]. VP has also been suggested to exert anti-proliferative effects and overcome the chemotherapy resistance in urothelial cell carcinoma and in esophageal cancer cells [208,209]. The YAP/TAZ–TEADs transcription complex constitutes the most attractive anti-cancer target in the Hippo pathway, even though there are several molecules that target the upstream effectors of the YAP/TAZ [210]. There are currently no available anti-cancer drugs for clinical practice for the PitNETs, but the inhibition of the downstream effector of the YAP/TAZ (AXL inhibitors and monoclonal CTGF antibodies) have been evaluated in clinical trials in several malignancies [211,212] and could be a therapeutic option for PitNETs in the future.

### 2.4. Wnt Pathway

Wingless/Int (Wnt) signaling is involved in pituitary organogenesis and controls the cell activity in the adult gland. The Wnt pathway has a pivotal role both in the differentiation of the pluripotent cells and in the proliferation of the mature pituitary cells, as well as in pituitary tumorigenesis. The most crucial component in the intracellular Wnt signaling pathway is β-catenin, an oncogenic protein encoded by the CTNNB1 gene. The Wnt proteins are the crucial regulators of this pathway, which interact with Frizzled (Fzd) receptor and facilitate the transcription of the cell proliferation and differentiation genes. In the inactive state (absence of the Wnt ligand), β-catenin is phosphorylated by the protein complex consisting of AXIN, glycogen synthase kinase-3β (GSK-3β), adenomatosis polyposis coli (APC), and casein kinase 1 alpha (CK1α), leading to its ubiquitination and degradation. In the active state (presence of the Wnt ligand), the regulatory complex axin/APC/GSK-3β/CK1α is inactivated by the disheveled (Dsh) protein, so β-catenin is not phosphorylated and enters the nucleus, acting as a transcription factor for the cell proliferation genes (cyclin D and c-Myc) [213] (Figure 2).

There is increasing evidence that Wnt signaling is implicated in the PitNets. It has been shown that Wnt4 was highly expressed in human pituitary tumors expressing GH, PRL, and TSH, all of which belong to the Pit1 cell lineage. Its presence was correlated with the Fzd6 expression, suggesting that the activation of the Wnt4/Fzd6 signaling contributed to tumorigenesis, but there was no change in the β-catenin distribution. β-catenin was localized only at the cell membrane in all the pituitary tumors and the normal pituitary glands. These findings indicated that the Wnt4/Fzd6 signaling was activated via a β-catenin-independent pathway [214]. Another study investigated 47 pituitary tumors in which β-catenin was localized in the cell membrane with no difference between the PitNETs and normal controls. Still, they found a high nuclear accumulation of the Wnt target genes Cyclin D1 and c-Myc in the tumor tissue, indicating a β-catenin-independent activation of the Wnt pathway [215]. Contrary to the previous studies, Semba et al. found a nuclear accumulation of β-catenin in 57% of the investigated PitNets, but they did not compare their findings to the normal pituitary gland [216]. A recent study showed the β-catenin immunohistochemistry at a lower percentage of β catenin in the membranes of the resistant lactotroph tumors compared to the normal glands, independent of the Ki-67 proliferation, but without a statistical difference in the percentage of nuclear and cytoplasmic accumulation. In the same study, a strong correlation of β-catenin with PRL and Cyclin D1 in the experimental lactotrophs and a downregulation of β-catenin and Cyclin D1 using the temozolomide treatment (TMZ) was detected [217]. Adding to the previous data, it was demonstrated the Wnt inhibitory factor 1 (WIF1) was significantly reduced in the pituitary tumors by the increased methylation of the WIF1 promoter [218]. In conclusion, even though the data are controversial in pituitary tumors regarding the β-catenin subcellular localization, there is no doubt that the Wnt signaling plays a crucial role in PitNETs. Therefore, there is an ultimate need to elucidate the precise mechanisms.

On the other hand, the activation of the canonical Wnt/β-catenin pathway is well established in ACPs, which have been linked with the activating mutations of the b-catenin encoding gene CTNNB1 and, subsequently, the Wnt/β-catenin pathway activation, which was first described by Sekine et al. in 2002 [219]. Several independent studies in ACPs identified the activating mutations of CTNNB1 and the nuclear β-catenin localization, suggesting the involvement of the Wnt pathway for the pathogenesis of ACPs [219,220,221,222,223]. This finding has been verified using ACP mouse models. Indeed, the CTNNB1 mutations in Rathke’s pouch (RP) progenitors using Hesx1^Cre/C^; Ctnnb1^lox(ex3)/C^ mutant mice caused large cystic pituitary tumors that histologically and radiologically resembled human ACPs [108,224,225].

Therefore, Wnt/β-catenin pathway could be a drug target candidate for the management of ACPs. Up to now there are many inhibitors of the Wnt/β-catenin pathway that are being investigated in both animal models and clinical trials in other types of cancer [226,227,228]. Nonetheless, as it is an important intracellular signaling pathway, there is rising concern about the possible detrimental side effects on the tissue homeostasis. Thus, it is not currently considered among the intervention strategies. For this reason, several ongoing clinical trials are studying the effect of the Wnt pathway inhibition in adults with solid tumors (ClinicalTrials.gov identifiers: NCT03901950, NCT02675946, NCT03447470); however, there are no ongoing clinical trials for CPs. The other targetable pathways downstream of the WNT/β-catenin pathway have also been identified. There is rising evidence the cystic and solid tumor components of ACPs have high levels of IL-6 and the treatment using tocilizumab, an IL-6 inhibitor, resulted in a significant cyst shrinkage in two patients for whom it was offered [229]. Based on this result, there is currently an open clinical trial using tocilizumab in children and adolescents with new or recurrent/progressed ACPs (ClinicalTrials.gov identifier (NCT number): NCT03970226).

Moreover, many studies have confirmed that the sonic hedgehog pathway, a significant regulator for organogenesis that cross talks with the Wnt pathway, is upregulated in mouse and human ACPs [110,224]. Unfortunately, treating the ACP mouse model as well as the patient-derived xenograft mice using vismodegib, an FDA-approved SHH pathway inhibitor, against the other tumors resulted in increased tumor cell proliferation, premature tumorigenesis, and reduced mouse survival [230,231].

Although ACPs do not carry mutations in the MAPK pathway, a novel study has recently demonstrated the involvement of the MAPK/ERK pathway in human and mouse ACP tumors, suggesting that the MEK inhibitors as potential drug candidates [232]. Interestingly, the MEK inhibitor trametinib has a reduced proliferation and increased apoptosis in vitro in the cell cultures of human and mouse ACPs [232]. Patel et al. verified these data in vivo, demonstrating the favorable efficacy of the MEK inhibitor binimetinib in a 26-year-old female with an ACP [233]. An ongoing trial in phase II has just started using binimetinib to treat pediatric patients diagnosed with recurrent ACPs, including patients who have undergone surgery and/or radiation therapy (ClinicalTrials.gov identifier (NCT number): NCT05286788).

Current knowledge has emerged about the interaction of the Hippo with the Wnt signaling in different tissues, namely two pathways that are crucial for development and homeostasis. However, various studies have reported that the YAP may have both positive and negative effects on the Wnt signaling. For example, the pYAP1 has been shown to have a positive influence on the Wnt pathway as it forms a complex with β-catenin, which drives the transcription of the antiapoptotic genes, including BCL2L1 and BIRC5 [234]. Interestingly, it has been suggested that the YAP/TAZ may be integral components of the β-catenin destruction complex, which are released from the complex and are translocated to the nucleus after the Wnt activation [235]. Adding to this finding, it has been recently shown using osteoblast lineage cells that the YAP interacts with β-catenin and stabilizes it into the nucleus to control bone homeostasis [236]. In contrast, another study reported that the YAP may have a negative effect on the Wnt pathway; when the hippo pathway is active, and the YAP/TAZ are in the cytoplasm, the TAZ binds to the Dsh protein and inhibits the Wnt signaling as well the β-catenin translocation to the nucleus [237]. These different roles of the YAP may be due to the different cell types, while the precise effect of this interaction remains to be elucidated.

## 3. Tumor Suppressor Genes/Oncogenes

### 3.1. Menin Gene

Multiple endocrine neoplasia type 1 (MEN1) syndrome is an autosomal dominant disorder with a high penetrance that is present in endocrine and non-endocrine tumors. Only 10% of patients are identified with de novo mutations. The patients are predisposed to the formation of the PitNETs, parathyroid hyperplasia, and gastroenteropancreatic neuroendocrine tumors (GEP-NETs) [238]. Parathyroid tumors are the most common in approx. 95% of patients, followed by GEP-NETs in approx. 40%. These include gastrinomas, insulinomas, pancreatic polypeptidomas (PPomas), glucagonomas, and vasoactive intestinal polypeptidomas (VIPomas). Anterior pituitary tumors occur in about 30–40% of patients and the most prevalent type is lactortroph tumors (28–80%), followed by NF-PitNETs (15–48.1%), somatotroph tumors (5–15%), co-secreting tumors (9.1%), and rarely corticotroph tumors (5%), depending on the different series [238,239,240]. Overall, MEN1 is responsible for less than 3% of patients with anterior pituitary tumors [241].

The causative defect is the germline heterozygous mutation *in the MEN1* gene, a tumor suppressor gene localized on chromosome 11q13 [242]. Until recently, more than 1200 germline mutations have been identified in the MEN1 gene. In the majority of patients, the tumor formation follows the Knudson’s “two hit model” having one germline mutation in the MEN1 gene while a loss of heterozygosity (LOH) or somatic mutations occurs in the MEN1 alleles of the tumor [243]. Menin is a nuclear protein with a ubiquitous expression, which is expressed differently from tissue to tissue [244]. The cytoplasmic expression, as well as in the cell membrane, has also been described but to a lesser extent. Menin can regulate the gene transcription either positively or negatively. Recent studies suggest that it may act as a scaffold protein that controls the gene expression and cell signaling [244]. Menin binds with the transcription factor JunD, one of the AP-1 transcription factors, and blocks its phosphorylation and activation from the c-Jun N-terminal kinase (JNK). Menin and JunD suppress the expression of the gastrin gene by binding to its promoter [244]. On the other hand, menin activates the gene transcription by forming complexes with the transcription activator mixed lineage leukemia protein 1 (MLL1), a methyltransferase which functions as an oncogenic co-factor to promote the gene transcription and leukemogenesis [244,245]. In addition, it can directly control the expression of the cyclin-dependent kinase inhibitors 1B and C (CDKIs), p27Kip1 and p18Ink4c, through the recruitment of the MLL. The loss of function of either the MLL or menin results in a downregulation of p27Kip1 and p18Ink4c and aberrant cell growth, suggesting that the cooperation of menin and the MLL plays a major role in menin’s activity as a tumor suppressor [246]. Moreover, recent studies suggest an interaction between menin and cyclin-dependent kinase 4 (CDK4), a regulator of the cell cycle during the G1/S transition. Thus, the downregulation of the CDKIs is responsible for the CDK4 activation in the pre-oncogenic menin-deficient cells [247].

It is of the utmost importance to recognize the patients with MEN1 and the affected family members for early screening and counseling [238,241]. MEN1 pituitary tumors are frequently macrotumors, occur at a younger age, and are considered to display a more aggressive behavior and resistance to treatment [248]. However, more recent studies have shown that these tumors usually respond well to medical treatment regimes, in line with PitNETs occurring in the general population [249]. The most recent Endocrine Society clinical practice guidelines for MEN1, published in 2012, recommend an initial screening for pituitary lesions in asymptomatic carriers at the age of 5 years [238]. Thus, this raises the necessitation for research into new treatment options. The inhibitors of the menin-MLL interaction hold promise for provoking new treatments, especially for their targeting of antileukemic effects. Currently, there are several ongoing studies in targeting molecules, especially due to their usefulness in the treatment of acute myeloid leukemia [250,251].

### 3.2. CDKN1B Gene

Not all patients with a MEN1-like phenotype harbor mutations in menin. About 10–15% have mutations in different genes and 3% of them carry germline mutations in the *CDKN1B* gene, classified as MEN4 [248]. The *CDKN1B* gene is a tumor suppression gene located on chromosome 12p13.1, encoding for the protein p27Kip1 (known as p27 or as KIP1) [252]. The protein p27 is a member of the CDKI family, which binds to the cyclin/cyclin-dependent kinase complexes, preventing the cell cycle progression. In most cases there are germline heterozygous nonsense mutations, which lead to a reduced expression of p27, thereby resulting in an uncontrolled cell cycle proliferation [253]. MEN4 patients usually exhibit parathyroid tumors and primary hyperparathyroidism. However, neuroendocrine tumors such as PitNETs, adrenal, and enteropancreatic tumors, testicular and papillary thyroid cancer, as well as non-endocrine tumors such as cervical carcinoma, colon cancer, and meningiomas, have also been reported [253,254]. Since the number of reported cases is quite low, it is not easy to assess the prevalence of each type of PitNET, their clinical behavior, and provide specific guidelines for treatment. To date, somatotroph and corticotroph tumors in MEN4 account for 10 and 5% of PitNETs, respectively, whereas lactotroph tumors are quite rare in contrast to the MEN1 patients [254]. The treatment is similar to the non-MEN4 patients. The first treatment option for pituitary MEN4 tumors is transphenoidal surgery. However, it is not always curative. If there is residual or recurrence of the tumor, radiation therapy is performed. A receptor-mediated pharmacological treatment (depending on the tumor subtype DAs or SSAs) is also used with a variable response [254].

### 3.3. CABLES1 (CDK5 and ABL Enzyme Substrate 1)

The *CABLES1* gene mapped in the chromosome locus 18q11.2 counteracts the cell cycle progression that is activated in the corticotroph cells in response to glucocorticoids in the adrenal–pituitary negative feedback. The loss-of-function mutations of this tumor suppressor gene leads to an uncontrolled cell proliferation in corticotropinomas [255]. The original description of the CABLES1 protein viewed it as an interacting partner and a substrate of the cyclin-dependent kinase-3 (CDK3) [256]. In addition, it stabilizes the regulators of the cell cycle, such as CDKN1A (P21), CDK5R1 (P35), and TP63, preventing their degradation [257,258]. Moreover, it seems to regulate the function of CDKN1B by contributing to its subcellular localization and degradation [257]. These pathways are often inactivated in Cushing’s disease. The patients are usually children or young adults who present cushingoid features or symptoms due to macroadenoma mass effects, silent corticotropinoma, and a high proliferative index. Most of the time, they are difficult to treat and exhibit a recurrence of their disease. In a reported series of patients, most of them required a second surgery with additional radiotherapy [257].

### 3.4. PitNETs Related to Succinate Dehydrogenase (SDHx) Mutations

The SDHx gene mutations are known for their implication in pheochromocytomas and paragagliomas tumor formation [259]. However, in 2012, Xekouki et al. described a patient with an acromegaly and concomitant presence of paragangliomas (PGLs) and pheochromocytomas (PHEOs) carrying a germline *SDHD* mutation while he exhibited loss of heterozygosity at the SDHD locus in the pituitary tumor, and increased transcription hypoxia-inducible factor α(HIF-1α) levels similar to the PHEO/PGLs [260]. Subsequently, the same group described the 3PAs syndrome characterized by the presence of the PHEOs and/or PGLs, and pituitary adenoma in the same patient [261]. Although the SDHx mutations are common in the 3PAs familiar cases (62.5–75%), they are quite rare in the sporadic setting of the syndrome (0.3–1.8%) [261,262].

The SDHx genes are tumor suppressor genes, encoding for the different subunits of the mitochondrial enzyme SDH, also named complex II or succinate:quinone oxidoreductase [263]. SDH is located in the inner mitochondrial membrane and has a critical role in the oxidative phosphorylation (OXPHOS) and tricarboxylic acid (TCA) cycles, two major mechanisms in the metabolism and energy production within the cells [264]. SDH consists of four subunits, SDHA-D. SDHA and B constitute the catalytic domain, which is extrinsic on the matrix side, while SDHC and D comprise the anchor subunits, which are intrinsic transmembrane proteins. The catalytic subunits catalyze the oxidation of the succinate to fumarate while the anchor subunits contribute to the transfer of the electrons from the succinate in the mitochondrial matrix to the ubiquinone in the inner membrane [264]. Several co-factors are required for the assembly and the placement of the SDH complex in its right location; two of them are the SDH assembly factors 1 and 2 (SDHAF1 and SDHAF2) [264]. The mutations in the SDH subunits, or as recently shown in the assembly factors, promote the accumulation of the succinate and the production of the reactive oxygen species (ROS). Both the succinate and the ROS induce the cataract of the pseudohypoxia reactions, i.e., they provoke hypoxic responses under normoxic conditions [265] and ultimately lead to the inappropriate activation of the HIFs [265]. Additionally, the succinate inhibits prolyl hydroxylase (PDH), which could have contributed to the degradation of the HIFs under normoxic conditions [264]. PDHs are further inhibited by the ROS production. As a result, the stabilized HIFs accumulate in the cell and induce tumorigenesis through the transcription of the nuclear genes involved in glycolysis, angiogenesis, and apoptosis, the prerequisites for tumor formation and expansion [263,265]. The increased expression of HIF1a in the pituitary tumor tissue detected in the original 3PAs case by Xekouki et al., established that hypoxia may be related to the pituitary tumor formation [260]. Furthermore, the accumulated ROS contributes to the oxidative damage of the DNA, which burdens the genomic instability [266]. Dénes et al. reported the presence of intracytoplasmic vacuoles in the pituitary tumor tissue with SDHx mutations, which might represent autophagic bodies [267]. This was further supported by the abnormal mitochondria found in the SDHB ^+/−^ mice [261]. Although this is still hypothetical, the co-existence of pseudohypoxia and autophagy in SDHx-mutated tumors might lead to chemo and radiotherapy resistance [268].

The PitNETs in the 3PAs are more common among familial cases and they are usually macroadenomas secreting PRL or GH, while less frequently, they can be non-functioning and secrete ACTH [269]. Most of the described cases required more than one type of treatment as they exhibited a more aggressive behavior and resistance to SSAs. Interestingly, the PitNETs in the context of the 3PAs were present at a younger age, in contrast to non-syndromic pituitary tumors, while the co-existence with the PHEO/PGLs was compatible with a more aggressive pituitary tumor, which implies a critical role of these tumors in the phenotype of the disease [267,269]. Following the cases described by Xekouki et al., [260,261,269], the other genes were implicated in the 3PAs phenotype, such as the Von Hippel Lindau, NF1, menin, RET, and recently, the Myc-associated factor X(MAX), a co-factor of the transcription factors Myc and MXD1, that regulate the cell proliferation, differentiation, and apoptosis [270,271,272].

The current knowledge of the molecular pathways involved in tumorigenesis in the 3PAs suggests new targeted therapeutic options for each molecular subtype. Research, especially in the field of SDHx-mutated PPGLs, raises new evolving therapies targeting the HIF/pseudohypoxia pathway, such as antiangiogenic therapies with a humanized vascular endothelial growth factor receptor (VEGF-A), monoclonal antibodies and TKIs, HIF-1a inhibitors, mTOR, and immune checkpoint inhibitors [273]. However, their use in SDHx-deficient PitNETs has yet to be determined.

### 3.5. DICER1, Ribonuclease III

DICER1 is a predisposition syndrome for the different types of tumors characterized by germline or mosaic loss-of-function (LOF) mutations in the *DICER1* gene mapped on the chromosome locus 14q32.13 [274]. It encodes a ubiquitously expressed endonuclease, a member of the ribonuclease (RNase) III family, required for the biogenesis of microRNA (miRNA) and small interfering RNA V (siRNA). However, the specific role of the *DICER1* gene in pituitary tumorigenesis is still under investigation [274,275]. The most characteristic tumor in DICER1 patients is pleuropulmonary blastoma (PBB), a rare, early childhood pulmonary mesenchyma tumor. The other tumors include cystic nephroma, Wilms tumors, ovarian sex cord-stromal tumors (OSCSTs), especially Sertoli–Leydig cell tumors (SLCTs), and childhood embryonal rhabdomyosarcomas (ERMS) [276]. Pituitary blastoma, a very rare embryonal aggressive pituitary tumor, can be part of DICER1 expressed with an ACTH-dependent hypercortisolemia (Cushing disease) and neuro-ophthalmopathy. Apart from surgery, polychemotherapy (cyclophosphamide, vincristine, methotrexate, carboplatin, and etoposide used in DICER1 patients) and adjuvant radiotherapy may be needed. However, the clinical experience with such tumors is very limited [277]. Recently, a cohort of pediatric patients (aged 7.8–16.3 years old) with corticotroph tumors and *DICER1* gene variants was described, suggesting a possible role for the *DICER1* gene defects in corticotroph tumorigenesis. Most of these patients had better outcomes [278].

## 4. Stem Cells in the Pituitary Gland and Tumorigenesis

In recent years, there has been convincing evidence of the presence of pituitary stem cells (PSCs), which are active in the embryonic and postnatal anterior pituitary gland [279]. PSCs are undifferentiated and can give rise to the three specific hormonal lineages characterized by the transcription factors, Pit1, Tpit, and SF1, which will differentiate into the hormone-producing cells (PRL, GH, TSH, LH/FSH, and ACTH) [280]. Similar to somatic stem cells, PSCs are capable of self-renewal and proliferation and seem to play a critical role in pituitary homeostasis and tumorigenesis [281,282].

In the last decade, a plethora of markers has been associated with the PSCs, demonstrating their clonogenic ability (S100β, SCA1, OCT4, NANOG SOX2, SOX9, CD44, CD133, NESTIN, PROP1, PRX1/2, GFRa2) [281,283,284,285,286,287,288]. SOX2-positive pituitary cells have been found to be predominantly grown as either adherent colonies or as free-floating spheres in cell cultures [224,289]. However, a small proportion of sorted SOX2 cells (1.5–5%) was capable of clonal expansion and self-renewal when cultured in stem-cell-promoting media, indicating the potential heterogeneity of the SOX2 population [224]. SOX2 has been shown to be expressed in all the cells in Rathke’s pouch, a primordium in the oral epithelium from which the anterior pituitary forms [290]. Postnatally, these positive cells are solely found in the marginal zone of the anterior pituitary, with some scattered cells forming groups in the parenchyma, where the SOX2 expression does not overlap with the differentiated hormonal markers. The cell-lineage-tracing experiments in vivo revealed that the SOX2 stem/progenitor cells persist into adult life and generate all the pituitary cell lineages while a proportion remains undifferentiated, suggesting that not all the SOX2 cells retain their stem cell capacity [281,282]. SOX2 is mostly co-expressed with SOX9 and partially overlaps with S100β along the marginal zone of the anterior pituitary, while the S100β-positive cells have been shown to have an enhanced clonogenic potential in vitro [281,282]. A growing number of studies demonstrated that most S100β-positive cells in the marginal zone and parenchyma of the adult anterior lobe were positive for SOX2 [282,289,291]. Subsequently, the findings above indicated that the SOX2/S100β cells were a representative type of adult pituitary stem cells. However, it is noteworthy that a recent study described the co-expression of CD9 in most of the S100β/SOX2-positive pituitary stem cells in adult rats. They found that this novel marker was involved in the vascularization of the anterior lobe as it was especially located in the tumor-induced neovascularization region in the rat lactotroph adenomas [292]. Recently, the Andoniadou group demonstrated that WNT/β-catenin regulates the SOX2 + PSCs for the postnatal pituitary expansion since SOX2 + stem cells secrete the WNT ligands that are essential for the proliferation of the neighboring lineage-committed progenitor cells [293].

Nowadays, it is well established that cancer stem cells (CSCs) stimulate tumor initiation, progression, recurrence, metastasis, and/or therapy resistance in different types of tumors. CSCs are characterized by persistent self-renewal and a multipotent differentiation capacity, representing a tumor-initiating cell population with intra-tumor heterogeneity [294]. Additionally, CSCs have high levels of plasticity with the ability to dedifferentiate. Similarly, CSCs have been identified in PitNETs. Several studies have isolated CSCs from human pituitary tumors with a clonogenic, sphere-forming potential in cultures that expressed pituitary-specific markers, such as Pit1, and markers of stemness, such as OCT4, Notch1 and 4, CD15, CD90, CD133, NESTIN, NANOG, CXCR4, and KLF4 [295,296,297,298,299,300]. Additionally, the regulatory signaling pathways that are essential for self-renewal and the differentiation of normal stem cells, such as Notch, Sonic hedgehog, Wnt, and Hippo are associated with cancer stem cells and pituitary oncogenesis as well [109].

Moreover, recent studies suggested that human pituitary adenoma stem cells (hPASCs) express DRD2, SSTR2, and SSTR5, whose activation using current treatment strategies such as DAs and SSAs seem to have promising results [296,297]. For example, Würth and his colleagues showed a decreased cell survival in hPASC cultures when incubated using the somatostatin/dopamine chimera BIM-23A760 [296]. Similarly, another study demonstrated that the DRD2 agonist BIM53097 and SSTR2 agonist BIM23120 had antiproliferative effects on both the spheres and tumor tissues in about half of the studied NF-PitNETs. In addition, the reduction in the proliferation ability of sphere-forming cells was confirmed by an increased CDKI p27 expression and a decrease in the cyclin D3 expression [297]. It is important to note that there was no difference in the frequency of the sphere formation between the NF-PitNETs that were in vitro resistant or sensitive to DRD2 and the SSTR2 agonists. However, the spheres that came from the tumors resistant to the DRD2 and SSTR2 agonists were larger compared to those derived from the sensitive NF-PitNETs [297]. Thus, these findings indicate that hPASCs are not responsible for the drug resistance to the standard treatments while they seem to be associated with their invasive behavior. Different studies have focused their attention on targeted PASCs therapy. A pioneering study revealed a tumor reduction in the xenografted somatolactotroph adenomas when treated using a γ-secretase inhibitor, which affects the stemness by Notch signaling interruption [301]. Another study demonstrated that AMD3100, a CXCR4 antagonist, reduced the AtT20 xenograft tumors growth of the PASCs [298]. Therefore, identifying the molecular profile of the PASCs provides interesting therapeutic targets.

## 5. MicroRNAs

MicroRNAs are short protein non-coding RNAs that act as regulatory proteins and control the post-transcriptional expression of specific genes through RNA interference and mRNA destabilization. They can induce a rapid degradation of the target messenger or inhibit its translation into a protein, and their expression can be regulated at different levels [302]. In 2005, their expression was described for the first time in the pituitary gland. Since then, several studies have shown that miRNAs are involved in many mechanisms regulating the pituitary hormone production, tumor formation, progression, and aggressiveness [303,304,305]. MiRNAs may play an important role in the pathogenesis and progression of PitNETs and may provide new molecular targets for their diagnosis and treatment.

It is estimated that miRNAs may control up to 50% of all the protein-coding genes [306]. Several miRNAs are found to be involved in cell proliferation and apoptosis through an interference with the different pathways. For instance, the miR-187-3p elevation seems to promote the cell cycle progression and inhibit the proliferation of pituitary tumor cells via the NF-κB signaling pathway [307]. Furthermore, the upregulation of several miRNAs (miR-17-5p, miR-20a, miR-106b, miR-21, miR200c, and miR-128) in pituitary tumors may inhibit the tumor suppressor signaling pathway PIK3/AKT, including PTEN, enabling a more aggressive behavior of these tumors [302,308]. On the other hand, another group of miRNAs (miR-132, miR-15a, and miR-16) has the ability to inhibit the cell invasion and metastasis in several PitNETs by targeting SOX5, rendering these miRNAs as potential therapeutic targets for more aggressive pituitary tumors [309]. Moreover, it was recently shown that the upregulation of miR-34a impairs the hormonal and antiproliferative response of the AIP + PitNETs to octreotide, implying that miR-34a may constitute another therapeutic target [310]. In addition, a recent study performed using a bioinformatic analysis revealed that among the different miRNAs that were identified, the overexpressed miR-149-5p and miR-99a-3p may have the ability to inhibit the progression of invasive PitNETs [311].

Altogether, these studies show that miRNAs may play a vital role in pituitary tumorigenesis and exhibit pituitary tumor characteristics and behavior. Since they were detected in biofluids and cell-free environments, they could serve as potential screenings or prognostic biomarkers in order to improve the diagnosis and response to the treatment of pituitary tumors and to follow or observe any early recurrence [312]. Moreover, the data shows that they may serve as novel drug targets, such as for epidrugs or antagomirs, since modulating the miRNA activity may restrain the tumor progression or weaken the symptoms associated with the aberrant hormonal secretion [305,313]. However, much remains to be investigated and understood in this promising field.

## 6. Conclusions

In conclusion, we thoroughly overviewed the current knowledge about the mechanisms of pituitary tumorigenesis, including the somatic and rarer germline mutations, in the genes pre-disposed to pituitary adenomas, as well as some the crucial molecules involving the MAPK and PI3K/Akt signaling, Wnt pathway, and lately the Hippo pathway. A summary of the current growth factor and pro-survival signaling pathways involved in pituitary tumorigenesis is illustrated in Figure 2. Moreover, we highlighted the involvement of the pituitary stem cells in pituitary pathogenesis and their molecular profile. Finally, as a tumor’s clinical behavior is affected by the genetic and molecular alterations discussed above, we spotlighted their clinical implication for the management of new therapeutic targets and new markers. With the rapid development of genome sequencing, elucidating the molecular mechanisms involved in PitNETs is of paramount importance for personalized medicine, especially for more aggressive, invasive, and drug-resistant tumors. To this end, future clinical studies and trials should focus on the genetic background and tumor molecular profile to improve the prognosis and survival in patients with PAs.

## Figures and Tables

**Table 1:** Molecular pathways and genes involved in the pathogenesis of the PitNETs.

Molecular Mechanisms	Protein (Gene)	Function	Syndrome	Mutation Origin	Clinical/Pathology Characteristics	Current Treatment Strategies
Gsa/protein kinase A/c AMP signaling pathway	G-coupled stimulatory protein subunit A (GNAS)	Oncogene, c-AMP pathway stimulation	McCune–Albright Sporadic	Mosaic Postzygotic	GH PitNETs, polyostic fibrus dysplasia, café au lait spots.	Surgery, SSAs—partial response
			-	Somatic	GH-secreting PitNETs in adults, less aggressive behavior, ACTH-, NF-secreting tumors rare.	Surgery, SSAs—most tumors seem to respond better. However, there are some controversial studies.
	Regulatory subunit protein kinase 1A (PRKAR1A)	PKA activity regulation	Carney Familiar/ sporadic	Germline	Pituitary GH/PRL hyperplasia, GH PitNETs, corticotropinomas, lactotroph adenomas, spotty skin pigmentation, PPNAD, myxomas, thyroid, testis, and ovarian tumors.	Surgery, SSAs, dopamine agonists. No systemic medical treatment developed according to the genetic defect or targeting the cAMP/PKA signaling pathway in the Carney complex.
	Aryl hydrocarbon receptor-interacting protein (AIP)	Tumor suppressor, co-chaperone protein	AIP-mutated adenomas Familiar/ sporadic	Germlline	Early onset of PitNETs GH, PRL, GH + PRL. NF- and ACTH- rare. Macroadenomas, pituitary apoplexy, aggressive.	Surgery, poor response to first-generation SSAs, and dopamine agonists. Better response to pasireotide. Interplay between AIP and RET pathway-RET inhibitors: potential new therapeutic approach for resistant tumors.Inhibition of CCL5/CCR5 pathway by maravirorik (experimental) as another therapeutic target.
	Orphan G-protein-coupled receptor (GPCR) protein (GPR101)	Oncogene, class A, rhodopsin-like orphan GPCR, coupled to Gs subunit, constitutive activation of the cAMP pathway	XLAG Familiar/ sporadic	Germline (females) Somatic (sporadic males)	Early childhood (<5 years old) onset gigantism due to GH-secreting or mixed GH- and PRL-secreting PitNets and/or hyperplasia, acanthosis nigricans, insulin resistance, increased appetite.	Surgery plus pegvisomat, radiotherapy. Resistance to SSA; potential new approach—therapeutic blockade of GHRH secretion (experimental).
MAPK/ERK pathway	Serine/threonine-protein kinase B-raf (BRAF)	B-Raf proto-oncogene, phosphorylate MEK and ERK1/2 kinases	Sporadic	Somatic	PCPs: GOF mutations BRAF V600E.	BRAF inhibitors as monotherapy/plus MEK inhibitors in cases of BRAFV600E mutant PCPs. Only one clinical trial for the treatment of BRAFV600E mutant PCPs: BRAF/MEK inhibitors (vemurafenib/cobimetinib) (NCT03224767).
					ACPs: BRAF V600E may coexist with CTNNB1-mutated ACPs.	
					ACTH-secreting. PitNETs: -Ras/ERK signaling activation -BRAF V600E in 16.4% of corticotroph tumors	MEK inhibitor (binimetinib), both in vitro and in vivo, and BRAF inhibitor (vemurafenib) in vitro inhibited corticotroph tumor cell proliferation and ACTH secretion.
PI3K/Akt pathway	Epidermal growth factor (EGF) receptor family of receptor tyrosine kinases (RTKs): the main TK target for PitNETs ErbB1 (EGFR) ErbB2 (HER2)	RTKs activate the MAPK/ERK and PI3K/Akt pathways leading to pituitary tumorigenesis	Sporadic	Somatic	Pit1 lineage-specific mTOR-activation leads to lactotroph adenomas in mice. Somatic mutations of PIK3CA in human PitNETs.	Everolimus: the only active mTOR inhibitor administered in patients with PitNETs. PI3K/mTOR inhibitors: a greater antiproliferative effect in vitro (no dual PI3K/mTOR inhibitor in clinical practice).
					HER2/ErbB2 induces PRL and tumorigenic effects in rat prolactin-secreting PitNETs. ErbB2 is mainly associated with aggressive and/or resistant prolactin-secreting PitNET in human studies.	Lapatinib, a dual EGFR and HER2 inhibitor, has a more influential role in prolactin-secreting PitNETs both in vitro and in vivo. A phase 2a clinical trial suggests that lapatinib may be a suitable treatment option for aggressive prolactin-secreting PitNETs.
					EGFR overexpression in ACTH-secreting PitNETs.	EGFR inhibitors (gefitinib and lapatinib), in both human and mouse corticotroph primary cultures.
	Ubiquitin-specific protease 8 (USP8)	Deubiquitinase controls the lysosomal trafficking and abundance of EGFR.	Sporadic	Somatic/one case of germline	GOF mutations of USP8 in 20–60% of ACTH-secreting PitNETs.	Treatment with pasireotide: correlation of USP8 mutational status with a higher SSTR5 expression.
HIPPO pathway	Yes-associated protein (YAP) Transcriptional co-activator with PDZ-binding motif (TAZ)	Oncogene, unphosphorylated nuclear YAP/TAZ act as co-activators to TEAD transcription factors.	Sporadic	Somatic	In SOX2 + pituitary stem cells in mice. In fetal and adult human pituitary. Increased expression in NF-PitNETs in humans.	No available YAP/TAZ inhibitors in clinical practice for PitNETs.
WNT pathway	b-catenin (CTNNB1)	Oncogene, unphosphorylated nuclear b-catenin acts as a transcription factor for cell proliferation genes.	Sporadic	Somatic	GOF mutations of CTNNB1 in ACPs.	WNT pathway is not considered among the intervention strategies for CPs. Tocilizumab, an IL-6 inhibitor in an open clinical trial for recurrent/progressed ACPs (NCT03970226).
Tumor suppressor genes	Menin (MEN1)	Tumor suppressor, nuclear protein with ubiquitous expression	MEN1 Familiar/ sporadic	Germline/ somatic	PitNETs—mostly PRL, followed by NF, GH—secreting rare pituitary carcinoma (macroadenomas, early onset, aggressive), parathyroid hyperplasia, and gastroenteropancreatic neuroendocrine tumors (GEP-NETs).	Surgery, SSAs, DAs, radiotherapy, temozolamide. Possible phenotype–genotype correlation.
	Cyclin-dependent kinase 1B/protein p27Kip1 (CDKN1B)	Tumor suppressor, CDK inhibitor-uncontrolled cell cycle proliferation	MEN4 Familiar/ sporadic	Germline	PitNEts (somatotroph, corticotroph), adrenal, enteropancreatic tumors, testicular, papillary thyroid cancer, non-endocrine tumors (cervical carcinoma, colon cancer, meningiomas).	Limited experience. Similar to that of non-MEN4 pituitary tumors.
	CDK5 and ABL enzyme substrate 1 (CABLES 1)	Tumor suppressor, counteraction of the cell cycle progression activated in corticotroph cells in response to glucocorticoids, regulation of the function of CDKN1B.	Sporadic	Germline	ACTH-secreting PitNETs or silent corticotropinomas, macroadenomas, children or young adults, cushingoid features, mass effect symptoms, high ki-67 proliferative index	Difficult to treat with a tendency to recure. Roscovitine (seliciclib), an inhibitor of the cyclin-dependent kinase cyclin E, effectively decrease the corticotroph cell growth—potential new therapeutic approach (experimental).
	Succinate dehydrogenase complex (subunits A,B,C,D) SDH assemply factor (SDHx)	Tumor suppressor, critical role in oxidative phosphorylation and tricarboxylic acid cycle.	3PAs Familiar/ sporadic (very rare)	Germline	PRL, GH, ACTH-secreting PitNets, aggressive macroadenomas. Pheochromocytomas and/or paragagglioma.	Surgery, poor responses to SSAs, new evolving therapies targeting HIF/pseudohypoxia pathway.
	DICER1 protein, ribonuclease (RNase) III (DICER1)	Tumor suppressor, endonouclease, member of the family ribonuclease (RNase) III, microRNA (miRNA) and small interfering RNA (siRNA)	DICER1 syndrome Familiar/ sporadic	Germline or mosaic loss-of-function (LOF)	Pituitary blastoma (ACTH-secreting) aggressive tumors, pleuropulmonary blastoma, cystic nephroma, Wilms tumor, ovarian sex cord-stromal tumor, embryonal rhabdomyosarcomas.	Surgery, polychemotherapy and adjuvant radiotherapy-limited experience.
Stem cells	SOX2/S100β pituitary stem cells	(1) Generate all pituitary cell lineages (2) Self-renewal (3) Clonal expansion	-	-	A representative type of adult PSCs.	-
	hPASCs express markers of stemness: OCT4, Notch1, 4, CD15, CD90, CD133, NESTIN, NANOG, CXCR4, KLF4	Sphere-forming potential in cultures that express pituitary-specific markers.	-	-	hPASCs express DRD2, SSTR2 and SSTR5.	Promising results by in vitro activation of DRD2, SSTR2, and SSTR5 using Das and SSAs analogues.
microRNAs	MiR-187-3p, MiR-17-5p, MiR-20a, MiR-106b, MiR-21, MiR200c, and MiR-128, MiR-132, miR-15a, miR-16, miR-34a, miR-149-5p, and miR-99a-3p	Short protein non-coding RNAs control the post-transcriptional expression of specific genes through RNA interference and mRNA destabilization; control up to 50% of all protein-coding genes	-	-	-	Potential novel drug targets. Act as epidrugs or antagomirs, since modulating miRNA activity may restrain the tumor progression or weaken the symptoms associated with aberrant hormonal secretion-experimental.

PitNETs, pituitary neuroendocrine tumors; SSAs, somatostatin analogues; GH, growth hormone; PRL, prolactin; PPNAD, primary pigmented micronodular adrenal hyperplasia; NF, non-functional; ACTH, adrenocorticotropic hormone; MEN, multiple endocrine neoplasia; XLAG, X-linked acrogigantism; GHRH, growth hormone-releasing hormone; HIF, hypoxia-inducible factor; PSC, pituitary stem cells; hPASCs, pituitary adenoma stem cells; GOF, gain-of-function; PCPs, papillary craniopharyngiomas; ACPs, adamantinomatous craniopharyngiomas; EGFR, epidermal growth factor receptor.

## Data Availability

Data are contained within the article.
